# Robust signalling entropy estimation for biological process characterisation

**DOI:** 10.1093/bib/bbaf269

**Published:** 2025-06-18

**Authors:** Ana Stolnicu, Nensi Ikonomi, Peter Eckhardt-Bellmann, Johann M Kraus, Hans A Kestler

**Affiliations:** Institute of Medical Systems Biology, Ulm University, 89069 Ulm, Germany; Institute of Medical Systems Biology, Ulm University, 89069 Ulm, Germany; Institute of Medical Systems Biology, Ulm University, 89069 Ulm, Germany; Institute of Medical Systems Biology, Ulm University, 89069 Ulm, Germany; Institute of Medical Systems Biology, Ulm University, 89069 Ulm, Germany

**Keywords:** signalling entropy, protein interaction networks, false-positive interactions, correction methods

## Abstract

**Motivation:**

Signalling entropy measures the uncertainty or randomness in the signalling pathways of a biological system. It reflects the complexity and variability of protein interactions and can indicate how information is processed within cells. Higher signalling entropy often indicates a more dynamic and adaptive state, whereas lower entropy may imply a more stable and less responsive condition. Estimating signalling entropy has become a valuable method for studying and understanding the complexity of biological processes. This measure has the potential to shed valuable insights into various phenomena, including the mechanisms behind cell fate decisions, drug resistance, and disease progression. To examine the molecular changes within a system, signalling entropy is quantified through the integration of expression measurements and protein interaction networks. Experimental and computational issues, such as false positives and additional noise, can all compromise the accuracy of protein interaction networks. Correction methods can be used to mitigate spurious results, correct for experimental bias, and integrate data from multiple sources. However, to date, the effect of such approaches on entropy calculations, together with the impact of different underlying networks, has yet to be evaluated.

**Results:**

Here, we investigate how the topology of distinct protein interaction networks can alter the entropy calculation. We examine the entropy derived from different protein interaction networks. Additionally, we systematically evaluate different correction strategies, outlining their benefits and drawbacks along with identifying the most effective approaches for specific types of data and biological scenarios. This protocol outlines how to optimize the reliability of entropy calculations and ultimately leads to a deeper comprehension of biological processes and disease mechanisms.

## Introduction

**Figure 1:**
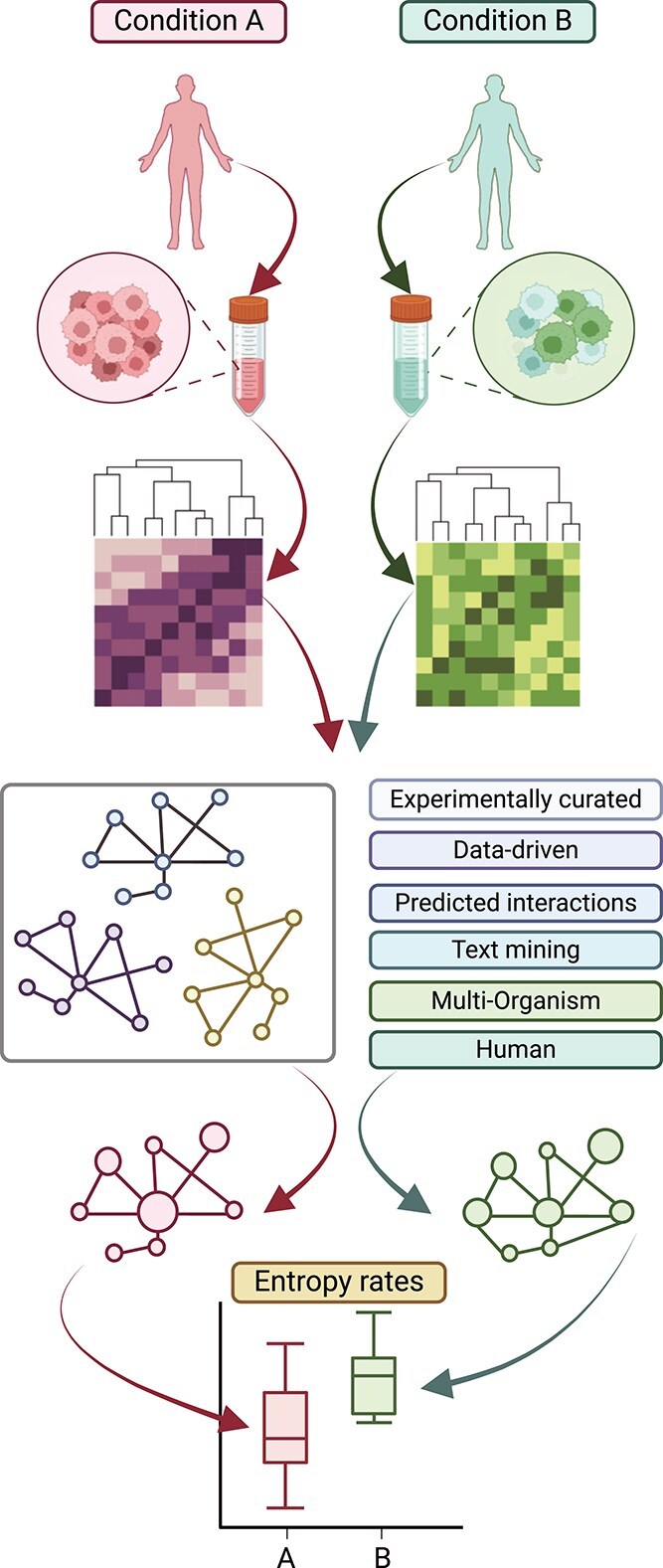
Overview of signalling entropy estimations. Starting from a biological process (e.g. cell differentiation, tumour progression), the corresponding biological samples are extracted for two or more conditions of interest. Expression data for each condition are inferred. To calculate signalling entropies, the expression patterns for each condition are integrated with protein interaction networks. These networks can be taken from different sources and filtered in a variety of ways. From this integration, signalling entropies are calculated and compared among different conditions.

The concept of entropy, adopted from physics and information theory, quantifies the degree of unpredictability or randomness in a given system [1, 2]. In molecular signalling networks, entropy can be used to quantify the complexity rate and diversity of signals flowing through the network [3, 4] (Fig. 1). Promising applications of entropy-based measures of signalling dynamics include the prediction of drug responses in cancer cells and the identification of critical regulatory networks in disease models [5, 6]. The application of entropy quantification has proven to be likewise advantageous in the examination of cellular differentiation mechanisms. This process has been associated with the descent of a cell through Waddington’s epigenetic landscape consisting of several valleys, each of which indicates an alternative fate for the cell [7]. The higher up the valley a cell is positioned, the higher the number of ways it can specialize in. In this scenario, the potential of a cell to assume distinct states can be quantified with the global signalling entropy rate. As such, a matured cell will have a distinct signalling entropy compared to a multi-potent one [8].

Differences in network entropy have been seen across tumour stages, suggesting that it may be linked to tumour initiation and progression [9, 10]. Moreover, a novel measure, the sample-perturbed network entropy (SPNE) [11], has been recently introduced to identify critical states in tumour progression data. The warning signals provided by SPNE during tumour progression have the potential to enhance prompt and suitable clinical interventions by early detection. By correlating expression patterns with a thorough protein interaction signalling network, Banerji *et al.* [8] established a measure to quantify the overall signalling entropy of cellular signal transduction networks. Pathway Commons (PC) [12], STRING [13], and the Biological General Repository for Interaction Datasets (BioGRID) [14] are just some examples of the various databases currently available that provide information regarding molecular interactions (MINTs). Despite their supposed reliability, the protein–protein interactions (PPIs) collected in these databases are gathered using various sources and methods, including yeast two-hybrid and mass spectrometry experiments. The effectiveness of these methods has been demonstrated in aiding the discovery of new PPIs and in evaluating the interactions between existing proteins [15–18]. Other approaches for identifying such relations comprise text mining [19] and computational modelling; nevertheless, confirmation of these interactions typically requires additional experimental testing. Regardless of integrating multiple techniques to get a more thorough understanding of PPIs, both experimental and computational approaches for detecting molecular relations are prone to high false positives (FPs) and false negatives (FNs). The inherent noise in the data, along with the complexity of biological systems, contribute to these rates [20, 21]. In yeast two-hybrid screenings, on which most interaction databases are constructed, FP rates are estimated to reach up to 64%, whereas FN rates range from 43% to 71% [22]. Additionally, it has been shown that the scale-free topology of the interaction network contributes to the increased signalling entropy observed in cancer samples compared to the healthy samples, providing further evidence for the entropy’s dependence on the characteristics of the protein interaction network (PIN) [23]. From a more general perspective, the topological properties of regulatory networks are fairly good predictors of dynamics [24].

Here, we intend to determine a potential network architecture showing minimum interference with the global signalling entropy changes across distinct stages in biological data. First, we review extensively the existing PINs, entropy calculation strategies, and their employment in different published studies dealing with entropy calculations. Then, we move into two specific case studies. We initially examine the influence of PIN topology changes and demonstrate how the selected PIN impacts the final entropy measure on both *in silico* generated and real experimental data. We show that the PIN used to calculate the signalling entropy can alter the statistically significant differences among the entropies resulting from the gene expressions of distinct samples. Furthermore, by employing correction techniques for FP interactions in the PINs, we evaluate and outline how the changes, dependent on the selected PIN, can be stabilized and harmonized. We conclude our problem-solving protocol by suggesting an effective strategy when calculating network entropies to avoid biases in its estimation that could affect the final interpretation and the biological conclusions of the experiments.

## Materials and methods

### Background on signalling entropy calculation

As introduced above, quantifying signalling entropy rates (SRs) requires combining a protein interaction network with multiple expression measurements, thereby rendering its accuracy contingent upon the integrity of both constituents. Classically, signalling entropy has been introduced as a measure to distinguish between cellular states throughout the process of cellular differentiation [8, 30] and was subsequently also used to investigate the mechanisms involved in the initiation and progression of cancer [8, 10, 31, 32], as well as tumour heterogeneity and metastasis [9, 33]. The integration of a PIN with expression data further relies on the mathematical approach for the calculation of the signalling entropy. Below, we review adopted approaches in selecting PINs and entropy calculations.

### PIN selection

As we are interested in capturing changes in signalling entropy resulting from variations in expression patterns (e.g. from multiple differentiation stages), it is imperative to minimize the impact of unreliable predicted interactions in the employed PIN, e.g. false-positive interactions. The choice of an appropriate experimental dataset is determined by the research question, but the choice of the PIN can also have a notable influence on the final outcomes [23]. The available PINs may vary in terms of the number of proteins involved, the number of interactions, the method used to detect protein interactions, and other related factors. An overview of the most commonly used PINs is given in Table 1. Here, we can observe that PIN databases can generally integrate interactions at different levels, in particular, protein and gene interactions. In addition, databases such as STRING also include predicted interactions via data mining and can integrate information from multiple databases to harmonise the existing information on protein interactions. This affects the final number of regulatory dependencies available in the PINs. Finally, different databases can integrate information from different organisms or limit their interactomes to single organisms, e.g. Human Protein Reference Database (HPRD) for human interactions. Thus, we could already observe a rather large heterogeneity in the construction and curation of interaction databases.

**Table 1:** Overview of PIN databases

Database	Protein interactions	Gene interactions	Predicted interactions	Integration of other databases	# Interactions	# Organisms
BioGrid [14]	✓	✓	X	X	2 039 483	71
STRING [13]	✓	✓	✓	✓	67 592 464	14 094
PC [12]	✓	✓	X	✓	2 424 055	Multiple
InnateDB [25]	✓	✓	✓	✓	829 948	3
HPRD [26]	✓	X	X	X	41 327	1
MINT [27]	✓	X	X	X	133 087	668
SIGNOR [28]	✓	X	X	✓	35 246	3
IntAct [29]	✓	X	X	X	1 194 594	3527
DIP [29]	✓	X	X	X	81 923	834

A list of commonly used PIN databases is provided, including BioGrid, STRING, InnateDB, the HPRD, the MINT database, the SIGnaling Network Open Resource (SIGNOR), IntAct, and the Database of Interaction Proteins (DIP). For each database, we provide information on experimentally validated protein interactions (e.g. two-hybrid assay), genetic interactions (e.g. co-expression), and predicted interactions (e.g. text mining of scientific abstracts). We also report whether each database provides information from other interaction databases. Finally, we include the number (#) of interactions and organisms documented for each database. For PC, the exact number of organisms is not given. Cells coloured in violet indicate the datasets used in the use cases of the problem-solving protocol. The data displayed in the table were queried on 31 May 2023.

We have continued to revise the PINs used in studies calculating network entropies. An overview is provided in Table 2. It is noteworthy that despite the predominant use of PC in previous entropy studies, there is still no consensus on the choice of protein interaction networks. In particular, one study focused on data-driven co-expression networks rather than relying on an interaction database. In addition, different filtering techniques were used in the different studies, resulting in widely variable numbers of nodes and edges within the interaction network. In particular, some studies focus their filtering on Kyoto Encyclopedia of Genes and Genomes (KEGG) annotations [34] or experimentally validated interactions. Others, instead, use the STRING database as a PIN and apply the internal confidence filter provided by the database, as well as filtering for human interactions only.

**Table 2:** Overview of the PINs used in previously published work on signalling entropy estimation

Publication	Year	PIN	# Proteins	# Interactions	Filtered	Filtering details
Teschendorff *et al.* [9]	2010	PC	7112	31 678	X	X
Banerji *et al.* [8]	2013	PC	8434	303 600	X	X
Teschendorff *et al.* [35]	2014	PC	10 720	152 889	✓	4 included databases
Banerji *et al.* [33]	2015	PC	8434	303 600	X	X
Menichetti *et al.* [31]	2015	PC	11 394	420 516	✓	KEGG annotation
Cheng *et al.* [10]	2016	InnateDB	13 579	113 473	✓	Experimentally validated
Teschendorff *et al.* [30]	2016	HPRD	8434	303 600	X	X
Zhong *et al.* [11]	2023	STRING	–	–		X
Gao *et al.* [36]	2022	Data-driven	–	–	–	–
Han *et al.* [37]	2020	STRING	11 451	65 625	✓	Human, confidence > 0.7
Liu *et al.* [38]	2020	STRING	–	–	✓	Removal of isolated nodes, confidence > 0.8

For each published work, the reference and year of publication are given. Along with the employed PINs, details on the number (#) of proteins and covered interactions are also provided. Finally, we include the information on whether the selected PINs were reported to have been filtered by the original authors. If this is the case, we describe the filtering approach in detail. ‘–’ indicates that the corresponding information was unavailable within the original publication. The 4 encompassed databases (in row 3) correspond to HPRD, NCI-PID, IntAct, and MINT.

### Strategies for the estimation of network entropies

**Figure 2:**
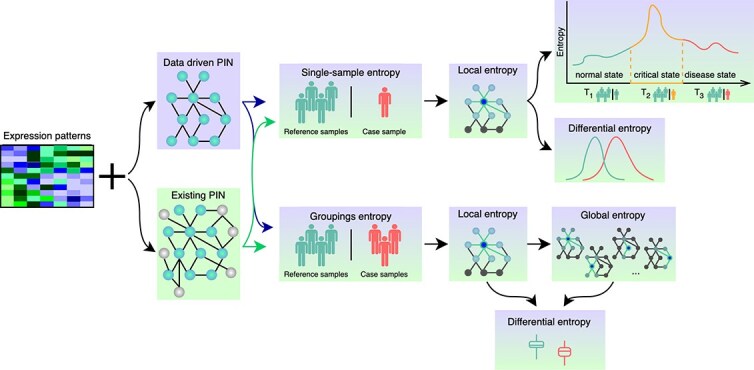
Outline of the available methods for the signalling entropy calculation. Generally, the signalling entropy in a biological system is measured by combining an initial set of expression patterns with either an existing or a derived protein interaction network. Based on the biological question and the available data, one can choose to either evaluate differences in single-case sample entropies or compare local or global entropies derived from distinct states.

As for the selection of the PINs, different studies have proposed a variety of strategies for calculating signalling entropies. An overview is provided in Table 3 and graphically depicted in Fig. 2. Likewise with selecting the experimental dataset, the methods for calculating the entropy of a system are dependent on the purpose of the study. One major characteristic distinguishing between the suggested methodologies regards the analysis of either single-sample entropies against a reference group or comparing groups of samples belonging to different classes. The single-sample approach is generally employed in detecting transition states, allowing the identification of involved biomarkers for individual cases. For instance, Zhong *et al.* [11] recommend examining the differences between the entropy computed on a progressive series of single samples and the entropy calculated on a set of reference samples to determine the key point of progression from a normal condition to a disease. On the other hand, one could evaluate the differences between the entropy rates obtained by two sets of samples representing two distinct states [8, 9, 23, 30, 33, 35, 39]. Another aspect to consider is whether it is more meaningful to analyse the local entropies [9, 10, 31, 35], defined for each node in the network, or the global entropy [8, 30, 33], describing the ambiguity of the whole system. While analysing the information flow around a single gene might be interesting in problems such as biomarker identification or understanding the dynamics of signalling molecules within a confined space; for investigating the overall complexity of a biological system and systemic responses to external stimuli, the utilisation of the global entropy might be beneficial. Regardless of the employed method, the overall objective is to utilise either node-entropy distribution analysis or global entropy differential analysis to investigate the differences between the entropy rates representing the involved groups.

**Table 3:** Overview of signalling entropy measures employed in previously published work

Publication	Local entropy	Global entropy	Entropy type
Teschendorff *et al.* [9]	✓	X	differential
Banerji *et al.* [8]	X	✓	X
Teschendorff *et al.* [35]	✓	✓	X
Banerji *et al.* [33]	X	✓	X
Menichetti *et al.* [31]	✓	X	single node
Cheng *et al.* [10]	✓	X	X
Teschendorff *et al.* [30]	X	✓	X
Zhong *et al.* [11]	✓	X	local SNE
Gao *et al.* [36]	✓	X	local SNE
Han *et al.* [37]	✓	X	local SNE
Liu *et al.* [38]	✓	X	local SNE

For each published paper, we report whether the local or the global entropy has been employed in the final analysis. Further information on the strategy for computing either the local or the global entropy is given.

### Calculation of the signalling entropy

Considering our review of PIN selections and entropy calculation strategies, we further defined our calculation strategy used for the case studies. First, considering the heterogeneous use of PINs, we decided to select PINs representing, at best, the variety of properties in interaction databases. Thus, we consider three PINs (PC, BioGRID, and STRING) together with their union and intersection, recalling the idea of combining information from multiple databases. Then, in order to compute the signalling entropy of a whole biological system, for Case Study 1, we considered the approach introduced by Banerji *et al.* [8] to measure the *equilibrium global entropy* and the *non-equilibrium global entropy* proposed by Teschendorff *et al.* [35]. Both measures are generated from the single node entropies, with the distinction that the first takes into account the non-normalized local entropies, whereas the second one uses the normalized ones. Briefly, the expression patterns of two neighbouring genes in the PIN, $i$ and $j$, are utilized to calculate the likelihood of the interaction occurring. According to the *mass-action principle* [40], the interaction intensity in a sample $s$ can be estimated by the product of the expression values $E_{is}E_{js}$, normalized in order to have $\sum _{j}p_{ij}=1$:


(1) \begin{align*}& {p}_{ij} = \left\{\begin{matrix} \frac{E_{is}E_{js}}{\sum_{k \in N_{i}}{E_{is}E_{ks}}}\text{,} & \textrm{if}\ j \in N_{i}\\ 0\text{,} & else \end{matrix}\right.\end{align*}

where $N_{i}$ denotes the set of neighbouring genes of gene $i$ in the PIN. The probabilities of interactions are then stored in an $n \times n$ probability matrix $P$, with $n$ representing the number of genes. The local entropy of the interaction distribution, $S_{i}$, for gene $i$, is defined to quantify the promiscuity level of its signalling within the sample:


(2) \begin{align*}& \mathrm{S}_{i} = - {\sum_{j \in N_{i}}{p_{ij}log(p_{ij})}}\end{align*}

As previously mentioned, to comprehensively capture the signalling flow of the entire network in a given sample, we analyse two global measures: the *equilibrium entropy rate*, $\tilde{S}_{R_{eq}}$, and the *non-equilibrium entropy rate*, $\tilde{S}_{R_{neq}}$. The first is defined with respect to the stationary distribution, $\pi = (\pi _{1}, \dots ,\pi _{n})$, of the stochastic matrix $P$ [8], represented by $\pi P = \pi $ and satisfying $\sum _{i=1}^{n}\pi _{i}=1\ \mathrm{with}\ \pi _{i}\ge 0 \text{} \forall i$.


(3) \begin{align*}& {\tilde{\textrm S}_{\textrm R_{\textrm{eq}}}} = {\frac{\sum_{i}{\pi_{i}S_{i}}}{M_{R}}}\end{align*}

where $S_{i}$ corresponds to the non-normalized local entropy and $M_{R}$ represents the maximum reachable entropy rate and depends on the network’s characteristics. The second corresponds to the mean of the normalized local entropies, as described in [35].


(4) \begin{align*}& {\tilde{\textrm S}_{\textrm R_{\textrm{neq}}}} = {\frac{\sum_{i}{\tilde{S}_{i}}}{n}}\end{align*}

here, $n$ corresponds to the number of computed local entropies, i.e. nodes in the interaction network and each $\tilde{S}_{i}$ is normalized to lie between 0 and 1 as:


(5) \begin{align*}& {\tilde{\textrm S}_{\textrm i}} = - \frac{1}{log(k_{i})} \sum_{j \in N_{i}}{p_{ij}log(p_{ij})}\end{align*}

with $k_{i}$ indicating the degree of node $i$.

Unless the *equilibrium entropy rate*, corresponding to a weighted average of the non-normalized local entropies, and largely dependent on the global topology of the network, the average of the normalized local entropies keeps the discriminatory power high while being independent on the network structure.

### PIN correction approaches

**Figure 3:**
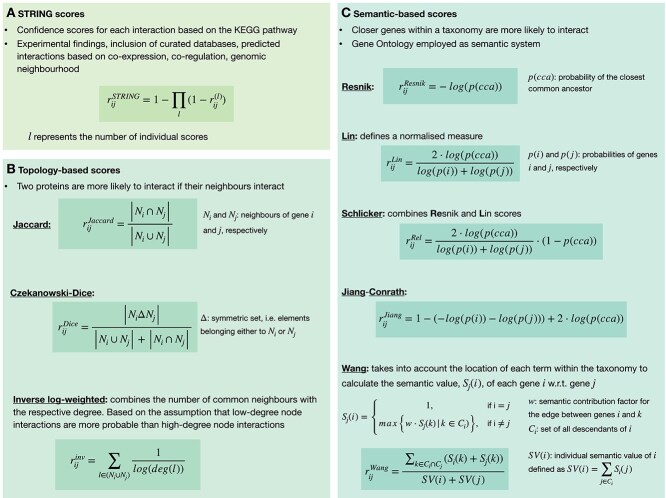
Correction methods for false-positive interactions are grouped by type, STRING scores (A), topology-based (B), and semantic-based (C), with a summary and mathematical details provided for each type.

We showed above that some entropy calculation studies applied filters to circumvent the issue of including false-positive regulations within the integrated PINs (Table 2). However, multiple methodologies have been devised to calculate the reliability scores of the interactions presented within the PIN. To date, no investigation has been conducted regarding the impact of diverse correction methods available. Thus, we further provided and tested different correction strategies applicable to PINs. In the following, we provide a brief overview of these procedures, also shown in Fig. 3. For additional details on each individual method, refer to the Supplementary Material (Section 1.3).

#### Topological scoring

Topological methodologies employ network characteristics, either at a global or local scale, encompassing factors such as neighbourhood, connectivity patterns, clustering coefficients, centrality metrics, or node degrees. These approaches aim to determine reliable and meaningful interactions. In accordance with these analysis strategies, the assessment of similarity between two proteins is contingent upon their respective interacting partners. The underlying premise of these metrics is that the likelihood of an interaction between two proteins is heightened when their neighbouring proteins also interact. The three similarity coefficients that we employed in this study to analyse the network properties comprise Jaccard [41, 42], Czekanowski-Dice [43] and the inverse log score [44].

#### Semantic scoring

The core element of semantic scoring techniques resides in incorporating information, such as functional annotations or Gene Ontology (GO) terms, to assess the biological relevance and reliability of protein interactions. These methods aim at leveraging the comprehension of the functional properties of proteins to furnish a quantitative measure of confidence for protein interactions. In our current investigation, we apply four semantic similarity measures, Resnik [45], Lin [46], Jiang-Conrath [47], and Schlicker [48], that rely solely on GO annotations to assess the similarity between terms. Additionally, we employed Wang’s approach [49], which integrates topological features of the protein interaction network in order to enhance the precision of the interaction scores.

#### STRING scoring

As a last correction approach, we used the scoring system provided by the STRING database. The combined confidence scores reflect the strength of each interaction based on various sources and methods comprising experimental and computational predictions, annotations from different databases, text mining, co-expression as well as transferred knowledge from other organisms. Notably, this score has already been employed recently by some authors (Table 2).

The R-package igraph, proposed by Csardi and Nepusz [50], is employed to calculate the topological reliability score. The semantic reliability score is measured by means of the R-package GoSemSim [51] and for the STRING scoring computation we employ STRING v.11.5 [13].

#### Inclusion of reliability scores into the entropy calculation

Upon incorporating expression profiles with the protein interaction network as weight factors, we implement the previously outlined correction techniques, following each of the corresponding methods. Essentially, a reliability score ranging between 0 and 1, is assigned to each interaction of the network and, by employing a series of thresholds, 0.1, 0.2,..., 0.9, we selectively filterout interactions with a score below the specified threshold. We keep the filtered network and proceed to compute the signalling entropy.

## Results and discussion

### Case studies

Our problem-solving protocol concentrates on two specific scenarios to delineate the optimal approach for achieving a stable calculation of signalling entropy. In the first case study, we examine an *in silico* yeast dataset to investigate the consequences of artificial interventions in protein interaction network structures and assess their impact on entropy calculations. This case study also serves as a proof of principle for the relevance of applying the subsequent correction methods.

In the second case study, we systematically assess the effects on entropy measures resulting from alterations of the underlying biologically motivated PINs. Subsequently, we employ correction techniques to ascertain the most effective combination of PIN and correction methodology conducive to obtaining a stable entropy measure. Ultimately, we outline a procedural framework for estimating signalling entropy, as detailed in Section 6.

### Case study 1 — yeast experiments

**Figure 4:**
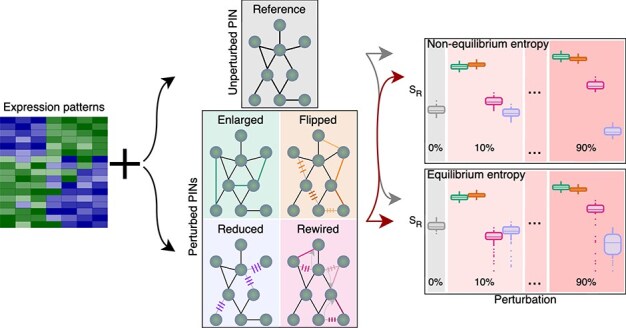
Workflow of the first case study, where the impact of artificial perturbations on entropy rates was tested by applying four types of changes, adding, flipping, rewiring, and removing edges, to a reference PIN, with perturbation levels from 10% to 90%, and entropy changes visualised for both equilibrium and non-equilibrium states.

In this scenario, we evaluated the impact of PIN alterations on the entropy values, as depicted in Fig. 4. To do so, we calculated the entropies resulting from networks in which we randomly added, deleted, flipped, or rewired interactions starting from our initial PIN and compared those results to the ones from our original unperturbed system. For this purpose, two entropy calculation strategies were employed respectively, equilibrium and non-equilibrium entropies. The yeast expression data was generated with the microarray data simulator GeneNetWeaver [52]. Signalling entropies were computed using the expression data and a PIN taken from the DREAM4 challenge [53]. The details of the experimental setup for this case study are presented in the Supplementary Material (Section 1.1).

**Figure 5:**
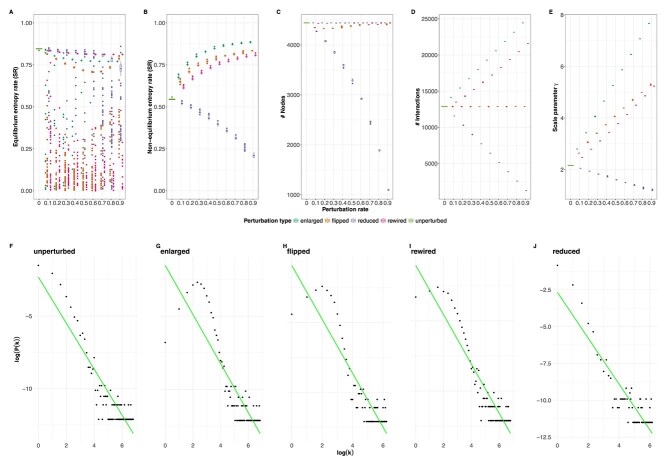
The effects of the network perturbations on equilibrium (A) and non-equilibrium entropy (B) are shown across perturbation levels, with higher variance in equilibrium entropy and increasing non-equilibrium entropy for all but removal; changes in network size (C, D), scale parameter γ (E), and degree distribution (F–J) reveal structural alterations and the loss of scale-free properties at high perturbation levels.

The results of the evaluation of the two employed measures, equilibrium and non-equilibrium entropy, are shown in Fig. 5A and B, respectively. For each experiment, we perturbed up to 90% of the initial interactome. For all four perturbation conditions, the calculated equilibrium entropies show outliers dropping in the entropy values. If, instead, the non-equilibrium entropies are considered, consistent trends can be observed for all perturbation settings. In this scenario, the inclusion, flipping and rewiring of edges lead to a gradual increase in entropy values, which saturates at about 80% of perturbed edges. The stagnating phenomenon might indicate that, after adding a certain percentage, the PIN attains a high level of connectivity causing any additional interactions to have minimal effect. On the other hand, the removal of edges leads to a general decrease in the entropy. Yet, the measured non-equilibrium entropies appear to be more stable without the above-mentioned entropy drops. We further examined the scale-free characteristic of each employed network. In Fig. 5E, depicting the changes of the scale parameter $\gamma $, we can notice that with low disruption rate (around 10%–20%) the values of $\gamma $ are ranged between 2 and 3, whereas as the perturbation levels rise, the values diverge from this interval. Thus, in contrast with the distribution of the unperturbed network (Fig. 5F) which exhibits an approximate scale-free structure, we can see in Fig. 5G–J that the degree distributions of the altered systems do not adhere to a power law with a rate of 60% of modified interactions. The degree distributions for all types and levels of perturbation can be found in the Supplementary Figs S3–S6.

The difference between the two calculation strategies is probably related to their intrinsic properties. The dips in the equilibrium entropy calculations could be related to the breakdown of the underlying largest connected component of the PIN on which the calculation is based, as shown in Fig. 5D. In particular, when removing interactions also, the number of nodes precipitates. Regardless of the perturbation type, we observe a consistent change in the entropy measures, which would affect the interpretation of the experimental results when comparing the signalling entropy within different conditions. Especially when edges are added, entropy calculations run the risk of saturating, therefore erasing differences between groups. This condition is relatable to the presence of false-positive edges within PIN databases. For instance, PINs constructed on two-hybrid screenings are reported to have a certain percentage (up to 64%) of false-positive edges [54] (in our scenario, added interactions). On these grounds, we tested the possibility of correcting entropy deviations by applying the STRING correction, to the network perturbed at 70% of added interactions. We could successfully notice a revert of the deviation effect on the entropy calculation at a threshold of 0.4. The result is shown in Fig. S1 of the Supplementary Material.

**Figure 6:**
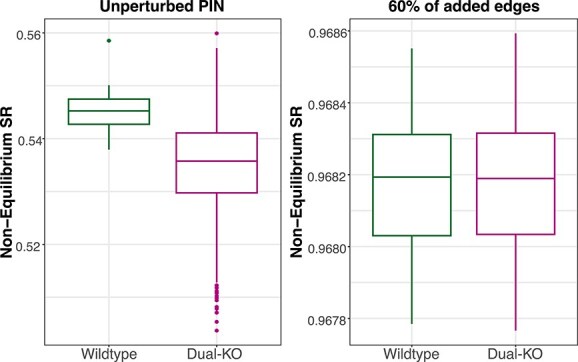
Network perturbations diminish discriminatory power: while wildtype and double knockout show significant entropy differences with the initial non-perturbed PIN, adding 60% new interactions neutralises this distinction.

Consequently, we tested the differences of the entropy values between our initial *in silico* data (referred as wildtype) and a derived group (referred as dual-KO), wherein each sample we knocked out a couple of genes from the strongly connected component. Figure 6A depicts the discriminatory power of the entropy values between the two conditions measured on the unmodified network, whereas in Fig. 6B we show how the wildtype and the dual-KO are not distinguishable when calculating the entropies based on a network comprising 60% of added interactions.

Considering our experiment on the yeast *in-silico* data, we could show that rather limited alternations can potentially affect the resulting entropy calculations. Based on this, we proceed in evaluating the alterations of entropy calculations within real expression data and PINs derived by different interaction databases. Considering the higher stability of the non-equilibrium entropy calculations, being less dependent on the network’s structure, and also its computationally lighter calculation, we further performed our experiments only with this measure.

### Case Study 2 — Physio-pathological experiments: differentiation & cancer

**Figure 7:**
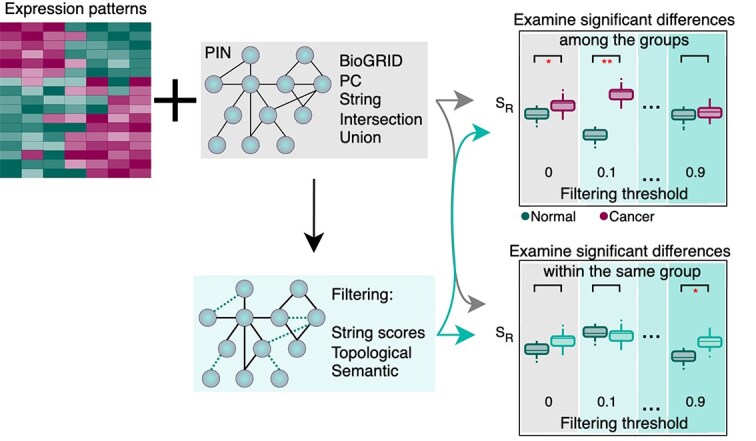
Workflow of Case Study 2, where PINs from PC, BioGRID, STRING, their union and intersection were combined with cancer and differentiation expression data to assess entropy variations between two classes, with results compared against those using semantic, topological, and STRING-based correction methods.

As a subsequent step, we proceed to the examination of real datasets and PINs. In this context, we assessed the efficacy of the entropy measures across three distinct datasets, concurrently evaluating and comparing the effect of various correction methods under different PINs (workflow depicted in Fig. 7).

We analysed three datasets concerning: pancreatic ductal adenocarcinoma (PDAC, GSE15471), human embryonic stem cells (hESC, GSE30652), and hepatocellular carcinoma (HCC, GSE6764). The datasets were selected to cover both developmental and cancer-related examples. Details of the dataset compositions are reported in the Supplementary Material (Section 1.2). For the underlying PINs, we considered BioGrid, PC, and STRING, respectively, together with their union and intersection. Among the databases available via PC, we used HPRD, IntAct, MINT, and NCI-PID, according to Teschendorff *et al.* [35]. In this way, we included three types of databases: PC, which contains mainly experimental evidence from protein interactions only, BioGrid, which contains experimental evidence from both protein and genetic interactions; and STRING, which contains both experimental and text-mined predicted interactions (see also Table 1). This choice is motivated by the fact that we want to cover different possible strategies in choosing the underlying PIN, as reflected in the variety of PINs presented in published work on entropy measures (see Table 2). Thus, based on our result in the yeast use case, we consider the PIN choice as a crucial first step in the entropy calculations. To assess the stability of our entropy measures, we evaluated the persistence of significant differences when comparing two groups within the datasets (healthy versus cancer or stem cells versus differentiated). This strategy aims to assess the ultimate interpretability of the measured entropies, which aim to detect significant differences between cellular entities. In this way, we exclude potentially irrelevant fluctuations in the entropy measures.

First, we calculated the significance of our entropy values based only on the different underlying PINs without applying any correction method. The results are shown in Table 4. It can be seen that, especially for the HCC dataset, the significance of the entropy differences between healthy and cancerous tissues are strongly influenced by the underlying PIN. In fact, when using the BioGRID dataset, the entropy differences are no longer significant. Instead of the three datasets, the most significant differences are observed for the hESC dataset. In fact, entropy measures were initially used to distinguish differentiation landscapes. Additionally, looking at the plotted entropy differences, we could observe that STRING and the union of the databases yield the highest entropy values (supplementary Fig. S2). This is because the two PINs are indeed the largest in size. We were able to show that also, in real datasets, the choice of PIN can strongly influence the final interpretation of the entropy differences between cellular conditions. This further motivates the use of correction methods.

**Table 4:** Significance analysis of the signalling entropy calculated on every combination of the three datasets with each initial PIN

Dataset	PIN **P**-value	Compared Classes
PDAC	\begin{align*} \left. \begin{array}{l} \textrm{BioGRID} \qquad\quad\!\! 3.30e^{-06}\\\textrm{PC} \qquad\qquad \qquad\!\! \!2.12e^{-07}\\\textrm{STRING} \quad \qquad 2.92e^{-07}\\\textrm{Intersection} \quad{9.33e}^{{-07}}\\\textrm{Union} \!\qquad\qquad 1.88e^{-07} \end{array}\right\} \end{align*}	Normal versus PDAC
HCC	\begin{align*} \left.\begin{array}{l} \textrm{BioGRID} \quad\qquad \!.15 \\ \textrm{PC} \quad\qquad\qquad\!\!\quad \!.02 \\ \textrm{STRING} \quad \qquad6.73 e^{-04} \\ \textrm{Intersection} \quad 1.90 e^{-04} \\ \textrm{Union} \quad \qquad \quad\! 8.27 e^{-04} \end{array} \right\} \end{align*}	Normal versus HCC
hESC	\begin{align*} \left.\begin{array} {l} \textrm{BioGRID} \quad\qquad 6.11e^{-17} \\\textrm{PC} \quad\qquad\qquad\!\!\quad 3.83e^{-17} \\\textrm{STRING} \quad\qquad\; 6.47e^{-06} \\\textrm{Intersection} \quad 2.81e^{-12}\\\textrm{Union} \quad\qquad\quad\! 4.47e^{-08} \end{array} \right\} \end{align*}	hESC versus Diff

In most of the cases, the differences are significant, excluding the HCC integrated with BioGRID.

We then evaluated the correction methods. The methods are divided, as mentioned above, into semantic, topological, and STRING correction methods. For each dataset, we applied each correction method to each PIN. In addition, we also calculated the significance level of differences within the same class by subsampling within the sample sets (e.g. healthy with healthy, cancer with cancer). The idea behind this evaluation is that a good correction method should preserve the significance levels between different classes but, at the same time, not generate false-positive significant differences within the same class.

**Figure 8:**
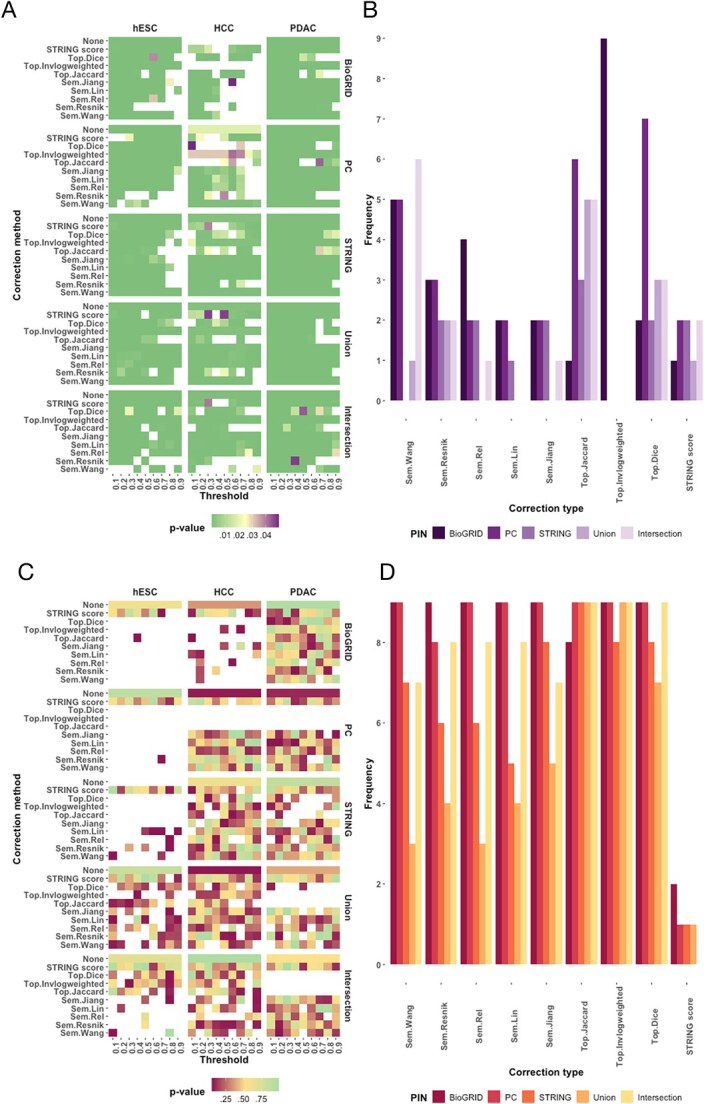
P-values for group differences, stem cells versus differentiated cells, or normal versus cancer are displayed in (A) across datasets and PINs with various correction thresholds (0.1–0.9); (B) shows the cumulative error counts (non-significant differences) for each filtering method; within-class comparisons (stem cells or healthy tissues) are detailed in (C); and the corresponding error frequency is indicated in (D).

To begin with the significant differences between the two classes, we can see that BioGRID, together with PC, are indeed the PINs that preserve fewer significant distinctions, also taking into account the correction methods (Fig. 8A). Instead, the STRING database, along with the union of the three databases, appears to be the one that best retains contrasts across groups. On the other hand, among the correction methods, the STRING filtering performs particularly well, especially in reversing the non-significant differences or increasing them, observed in the HCC dataset for the BioGRID PIN, shifting the result at almost every chosen threshold (Fig. 8A). Overall, we could observe that in general, considering all datasets and thresholds, the STRING filtering performs quite well in preserving the significant difference between classes. The worst correction method seems to be the Jaccard-topological, followed by the Wang-semantic. Considering the underlying initial PIN, instead, BioGRID is the worst initial PIN despite the use of correction methods, while STRING and the union of PINs perform better. Finally, the combination of STRING filtering and STRING/union as PINs gives the best results in terms of a lower number of errors (producing non-significant differences), as we can see in Fig. 8B.

We further evaluated the opposite scenario, considering the potential generation of false-positive significant values coming from PIN corrections. The experiment was performed by sampling within both classes separately. Nevertheless, we consider it more relevant not to induce false-positives within healthy/stem tissues. Instead, we cannot exclude the possibility of significant results when subsampling the cancer samples due to the potential effects of tumour heterogeneity and staging. For this reason, Fig. 8C depicts the $P$-value distributions within the healthy/stem classes. Here, we could observe that only the hESC dataset depicted a significant result within the stem population for the STRING database. Instead, all other PINs for unperturbed measures successfully retrieved non-significant differences within the same class. Interestingly, we could observe an extensive insurgence of false-positive significant results when applying either the topological or the semantic corrections. This phenomenon is particularly observed within the hESC dataset, the HCC one for the BioGRID PIN, and the PC, union, and intersection, especially for the topological corrections. Considering now the cumulative ability of the specific correction methods in preserving non-significant differences, we could again confirm that the STRING filtering is the one inducing the least switches from non-significant to significant differences. Considering instead the underlying PINs, again BioGRID and PC perform worst among the different correction methods. Consistently, STRING and union perform better for all corrections.

### Protocol of entropy stabilisation

We could show that the choice of the underlying PIN does affect the interpretation of entropy differences among different biological conditions. Considering the results of our problem-solving protocol, we summarise in the following a procedural strategy to ensure stable entropy calculations within expression patterns and PINs.

Therefore, among the non-corrected underlying PINs, we suggest using STRING or the union of different PINs. However, considering the heterogeneity of the results, we also strongly advise employing a correction method. Among the ones tested, the scores provided by STRING showed by far the best properties on stabilising entropy calculations. Thus, we suggest the use of the STRING testing threshold above 0.6 at least. Ultimately, when looking to evaluate any experimental design involving another PIN, we encourage utilising a filtering method among STRING or semantic Lin or Jiang, which demonstrated nearly comparable results. This indicates that most reliable PPIs are those that facilitate the selection of biologically trustworthy interactions, beginning with the largest possible set of interactions and nodes. Additionally, our research confirms that biological reliability filters outperform other correction methods.

## Conclusion

Using network entropy as a measure for the understanding of information complexity within different biologically motivated conditions is becoming increasingly common. This approach can also help identify pathways and targets of interest within the considered PINs. However, very limited, if not any, attention has been paid up to now to the stability of this measure when considering different underlying PINs. In this problem-solving protocol, we first extensively studied the effects of PIN perturbations on different entropy calculation strategies. Secondly, we moved to the analysis of real expression data in the context of physiology and cancer biology. By employing different PINs derived from widely used interaction datasets, we could indeed show that the choice of the PIN can strongly affect the final interpretation of entropy changes. Thus, we benchmarked various PIN correction methods aimed at stabilising global entropy changes among different biological classes. Our results indicate that the combination of the STRING PIN and correction is the most stable one for evaluating entropy changes. Based on these findings, we were able to propose a protocol for reliably accessing entropy changes within biological network data.

**Key Points**

- Network signalling entropy is a widely used measure to evaluate alterations within interactomes during physiopathological processes.
- The choice of underlying PIN can extensively affect the entropy calculation, and thus the final conclusions drawn from experiments.
- Correction methods for false positives should be considered when approaching entropy calculations.
- combining either the STRING database or the union of different PINs together with the STRING filtering provides reliable entropy measures.

## Supplementary Material

Supplements_bbaf269

## Data Availability

The data used in this work is all public.

## Code availability

The code to reproduce the analyses is available upon Git repository (https://github.com/sysbio-bioinf/signallingEntropy)
