# Supplementary material for: Robust signalling entropy estimation for biological process characterisation
Source: Brief Bioinform. 2025 Jun 18;26(3):bbaf269. doi: 10.1093/bib/bbaf269 (PMC12204201; doi:10.1093/bib/bbaf269)
Supplement: Supplements_bbaf269 [file supplements_bbaf269.pdf]

Robust signalling entropy estimation for biological process characterisation

Supplementary Information

Ana Stolnicu[1,+], Nensi Ikonomi[1,+], Peter Eckhardt-Bellmann[1], Johann M. Kraus[1,+], Hans A. Kestler[1,+]

[1] Institute of Medical Systems Biology, Ulm University, Ulm, 89081, Germany
[+] These authors contributed equally

1 Supplementary Methods

1.1 Experimental set-up Case Study 1

In the first case study we are interested in analysing the trend of the entropy measures under different perturbations of the interaction network. To this purpose *in silico* expression data was produced and utilised in combination with the integrated yeast protein interaction network.

The simulated yeast dataset has been created through the utilization of the microarray data generator GeneNetWeaver [13] (version 3.0), by taking in input the default simulation settings. It consists of 25 independent samples each containing 4441 randomly assigned expression values, we refer to this dataset as *wildtype.* In order to investigate the alterations in the entropy values after gene expression changes, a *dual knock-out* dataset has been generated from our initial simulated data. To this purpose the strongly connected component has been extracted, resulting in 60 genes and 207 interaction. Then each pair of these genes have been set to 0 in a randomly chosen sample, among the initial 25, resulting in a double knock-out dataset with 1770 samples and 4441 genes.

The Protein Interaction Network (PIN) utilised for the yeast case study was provided by the DREAM4 Challenge [6] comprising 4441 proteins and 12864 interactions.

At first the simulated expression data has been combined with the given unperturbed PIN and the obtained signaling entropy values were used as a reference for the subsequent results from the network modifications. We evaluated four types of interaction changes in the PIN: added, removed, flipped and rewired interactions. For every perturbation type 10 simulated networks were generated for each percentage in the series between 10% and 90%, by randomly modifying the existing interactions.

The scale-free property for each network has been verified and the corresponding degree distributions are displayed: Supplementary Figures 3, 4, 5, 6 show the increasing divergences of networks' structure from the scale-freeness for each alteration, enlarged, flipped, reduced and rewired, respectively, at each rate.

The individual resulted networks were then enriched with the yeast expression values for the entropy calculation and compared with our reference results.

Furthermore we were interested in investigating the impact of artificially altered networks on the significant differences between of entropies calculated for different groups. In this context we utilized the Wilcoxon rank sum test to compare the differences between entropies retrieved from the wildtype and the knock-out data first incorporated with the original PIN then integrated with the enlarged network with 60% of interactions.

1.2 Experimental set-up Case Study 2

The three real datasets we evaluate are publicly available in the GEO database. The Pancreatic ductal adenocarcinoma dataset (PDAC, Accession number GSE15471) describes a total of 78 samples divided into 39 pancreatic tumour and 39 normal samples with 43153 gene expression values [3]. From the human embryonic stem cells (hESC, Accession number GSE30652) we extracted 107 samples among the stem cells and the 32 somatic samples representing distinct tissues, for each 22819 gene were measured[10]. Finally we considered the expression profiles of the Hepatocellular carcinoma (HCC, Accession number GSE6764) comprising 75 samples representing 40 normal and preneoplastic lesions and 35 neoplastic stages[16].

To build our PINs we considered three existing databases, including STRING (`https://string-db.org/`), Pathway Commons (PC) (`https://www.pathwaycommons.org/`) and Biological General Repository for Interaction Datasets (BioGRID) (`https://thebiogrid.org/`), together with their union and intersection. For each we retained non-redundant interactions and unique nodes with an ENTREZ gene ID annotation. After the initial filtering step STRING (v11.5 downloaded on 9.12.2022) resulted in a network containing 18311 genes and 11035443 interactions. The interaction network obtained from PC was reduced to include the Human Protein Reference Database (HPRD) [11], the Interactome (IntAct) (`https://www.ebi.ac.uk/intact/home`), the Molecular Interaction Database (MINT) (`https://mint.bio.uniroma2.it/`) and the National Cancer Institute Nature Pathway Interaction Database (NCI-PID), resulting into a network with 10699 nodes and 756037 connections. The network derived from BioGRID (4.4.217, accessed on 30.12.2022) comprises 25087 proteins and 649026 interactions. Next when constructed the union of the three databases we obtained a structure with 11907839 relations and 26686 genes, whereas the intersection was composed by 41899 interactions and 6739 nodes.

1.3 Correction methods

STRING confidence scores:
The STRING database provides a set of confidence scores associated with each

interaction that indicate the perceived probability of being accurate. These grades are based on different criteria, for instance, neighbourhood, co-occurrence, co-expression, experiments or text-mining.

Topological scores:
we evaluate three approaches based on the network structure to compute the reliability score of an interaction r_{ij} between genes i and j, with $\mathcal{N}_i$ and $\mathcal{N}_j$ the set of first level neighbouring genes of i and j respectively, are defined as follows:

The Jaccard score [7, 17]:

$$r_{ij}^{Jaccard} = \frac{|\mathrm{N}_i \cap \mathrm{N}_j|}{|\mathrm{N}_i \cup \mathrm{N}_j|} \tag{1}$$

The Czekanowski-Dice score:

$$r_{ij}^{Dice} = \sum \frac{|\mathrm{N}_i \bigtriangleup \mathrm{N}_j|}{|\mathrm{N}_i \cup \mathrm{N}_j| + |\mathrm{N}_i \cap \mathrm{N}_j|} \tag{2}$$

where $\bigtriangleup$ indicates the set of components that can occur either in N_i or to N_j but not to both [4].

The inverse log frequency is calculated based on the assumption that interactions connecting low degree nodes have a higher probability than those connecting high degree nodes, as such, it combines the number of shared neighbours and their degrees deg [1]:

$$r_{ij}^{inv} = \sum_{l \in (\mathrm{N}_i \cup \mathrm{N}_j)} \frac{1}{log(deg(l))} \tag{3}$$

Semantic scores:
We evaluated five approaches based on a chosen taxonomy, which provides semantic information. The idea behind these methods is that genes that are more similar to one another in a certain taxonomy would also interact more frequently. For this purpose we use Gene Ontology (GO) [2, 5] which is grounded on compartment classification and is suited for this task since genes that are not located in nearby compartments are presumably not likely to interact. One of these approaches also considers the taxonomy's topology [15], while the other four are solely dependent on a term's information content [12, 9, 14, 8]. The negative log probability $-log(p(i))$ can be used to represent the information content of a term i. Here the probability of a term $p(i)$ can be estimated by the frequency $p(i) = \frac{freq(i)}{|T|}$ where $|T|$ represent the terms within a taxonomy. The frequency of i, $freq(i)$, corresponds to the sum of the occurrences of i itself and the occurrences of its child terms, as $freq(i) = freq(i) + \sum_{j \in C_i} freq(j)$, where C_i denotes the set of descendant terms of i. The information content of the nearest common ancestor (cca) sets an upper bound and it is utilized as a similarity measure between two terms i and j.

Resnik [12] directly defines the similarity rate as:

$$r^{Resnik}(i,j) = -log(p(cca)) \tag{4}$$

Lin [9] proposed a normalised measure:

$$r^{Lin}(i,j) = \frac{2 \cdot log(p(cca))}{log(p(i)) + log(p(j))} \tag{5}$$

Schlicker et al. [14] combined the previous ones resulting in:

$$r^{Rel}(i,j) = \frac{2 \cdot log(p(cca))}{log(p(i)) + log(p(j))} \cdot (1 - p(cca)) \tag{6}$$

Jiang and Conrath [8] derived a similarity measure by evaluating the connection strength between a descendant and its parent as the difference of their information content:

$$r^{Jiang}(i,j) = 1 - (-log(p(i)) - log(p(j))) + 2 \cdot log(p(cca)) \tag{7}$$

Wang et al. [15] finally, introduced a measure that considers each term's position in the taxonomy. The directed acyclic graph (DAG) of a term i is defined as $DAG_i = (i, C_{i,E_i})$ with C_i corresponding to the set of words descending from i and E_i set of edges. For every term i relative to term j, the semantic value $S_j(i)(t)$ is computed based on the position as:

$$S_j(i) = \begin{cases} 1, & \text{if } i = j \\ max(w \cdot S_j(k)|k \in C_i, & \text{if } i \neq j \end{cases} \tag{8}$$

The overall semantic value of i is calculated as the sum of the individual semantic values $SV(i) = \sum_{j \in C_i} S_i(j)$ and similarity between two terms i and j is defined as:

$$r^{Wang}(i,j) = \frac{\sum_{k \in C_i \cap C_j} S_i(k) + S_j(k)}{SV(i) + SV(j)} \tag{9}$$

2 Supplementary Figures

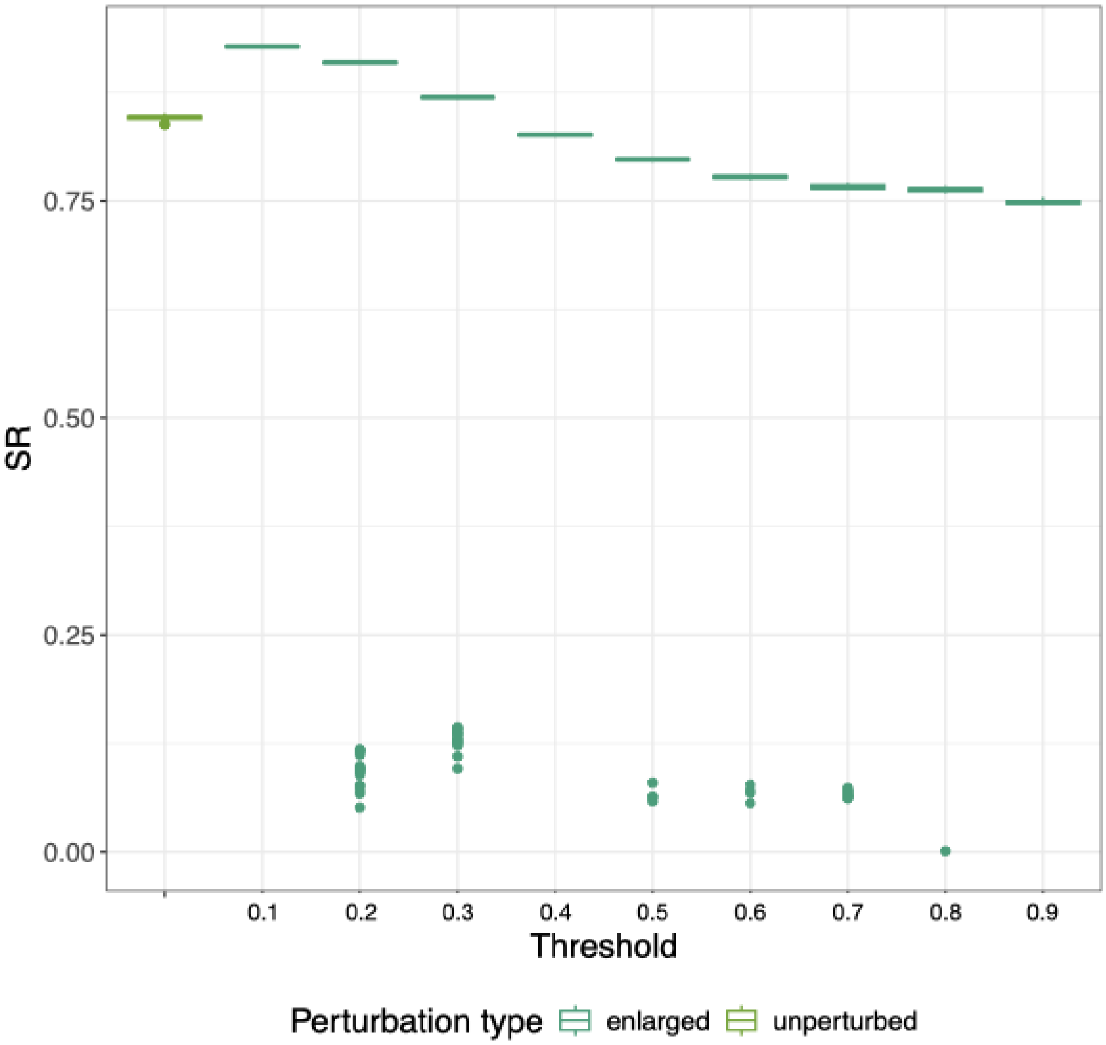

Figure 1: Entropy rates, calculated after applying the STRING filtering with different thresholds on a network modified with 70% of newly added interactions.

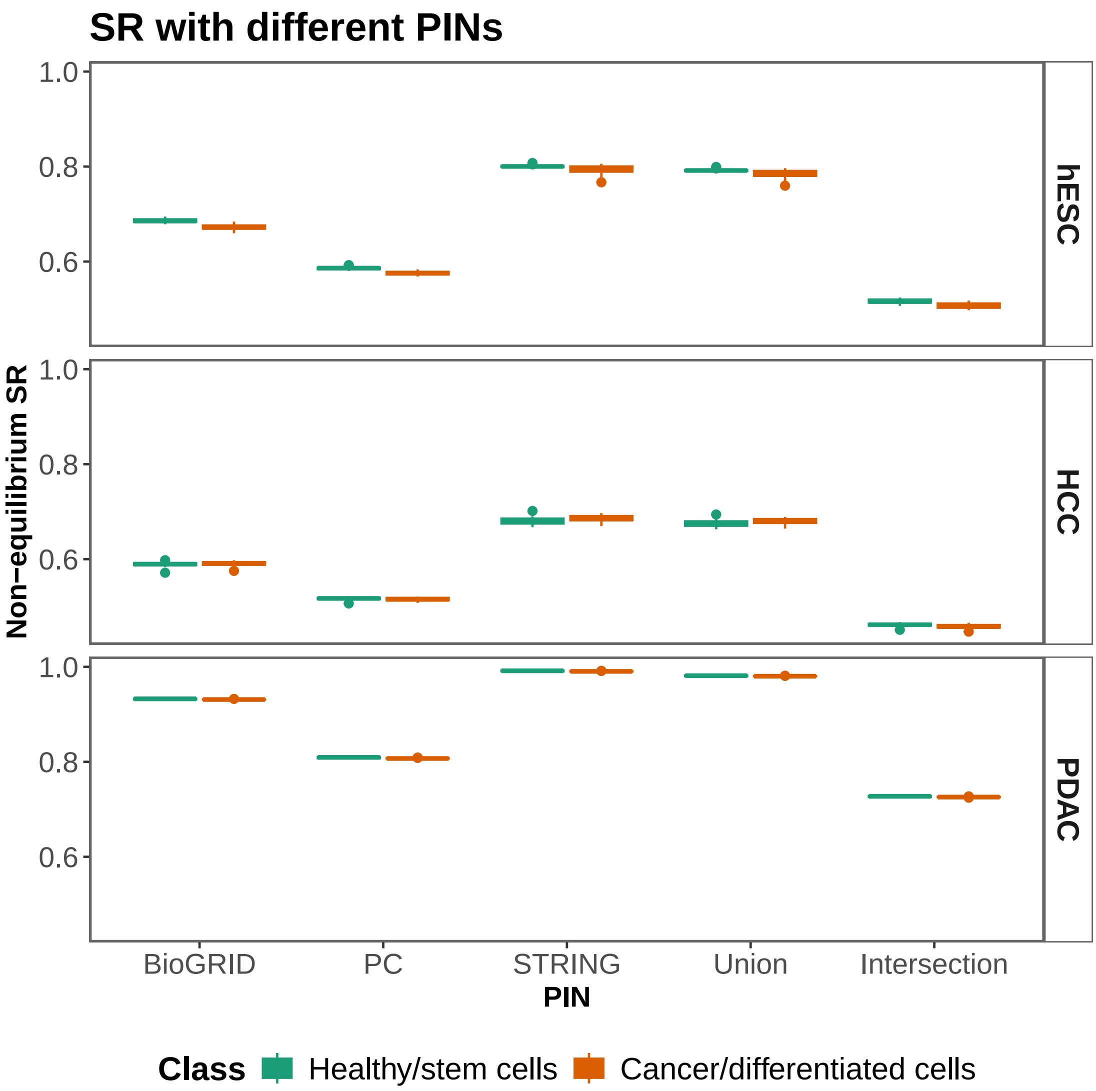

Figure 2: Changes in the signalling entropy values when using different PINs in combination with the three considered expression data

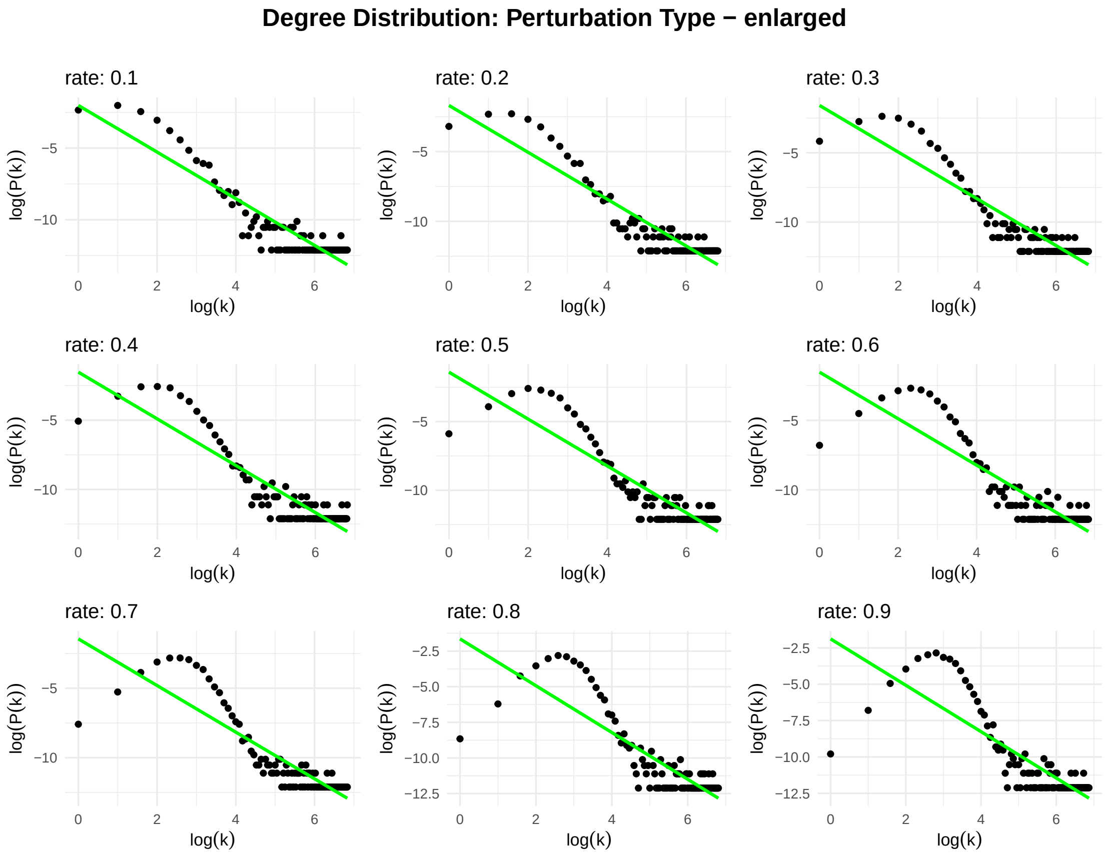

Figure 3: Distribution of degrees in the enlarged networks.

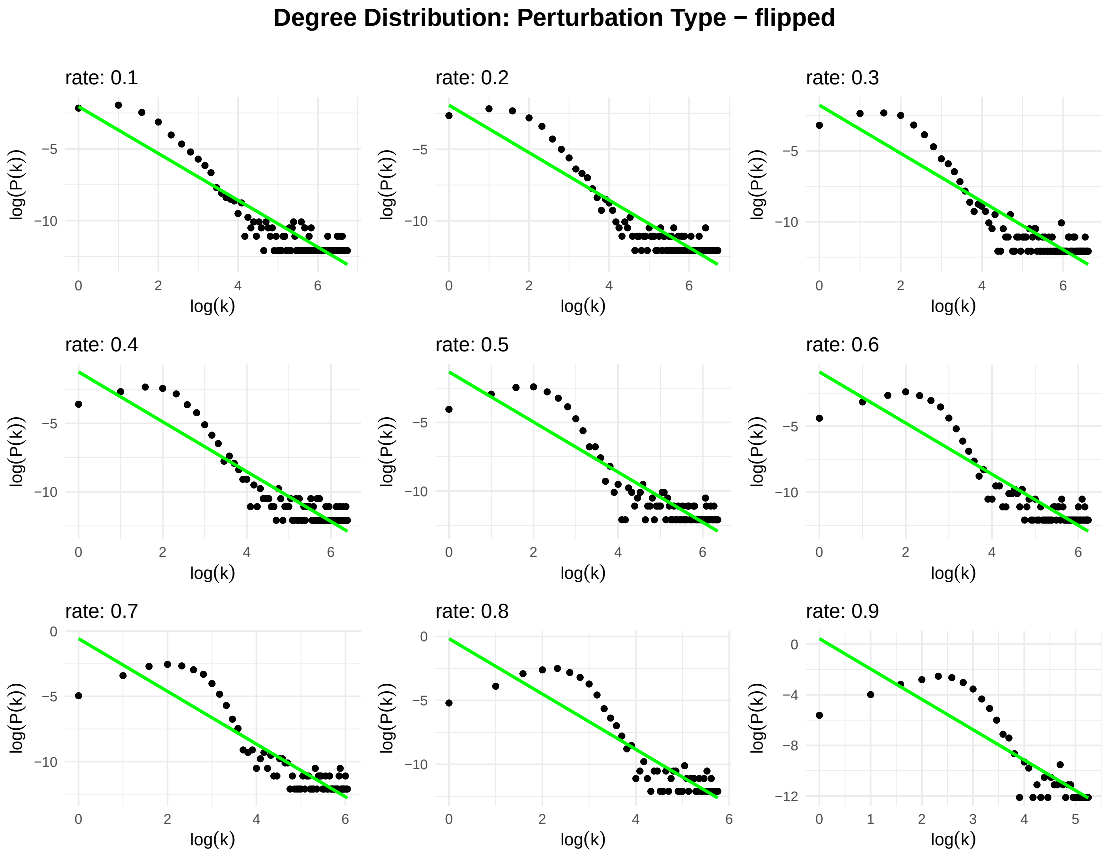

Figure 4: Distribution of degrees in the networks with flipped interactions.

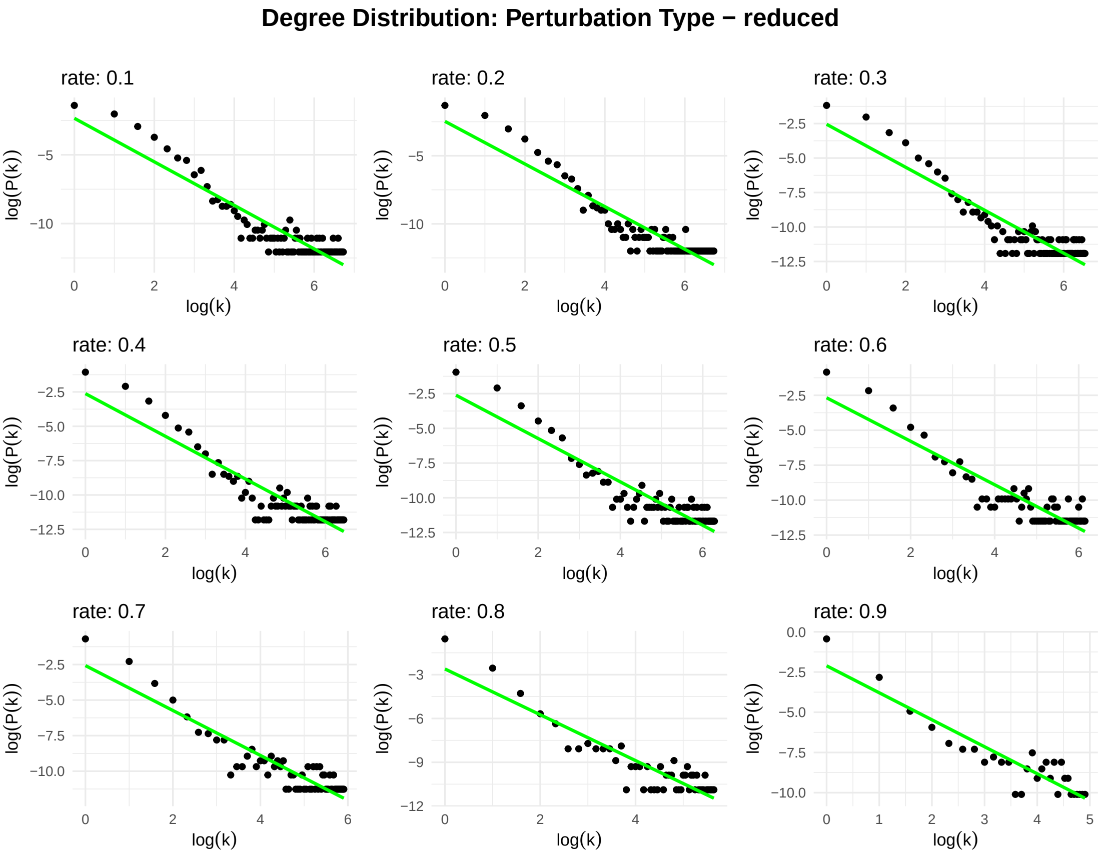

Figure 5: Distribution of degrees in the networks with reduced number of interactions.

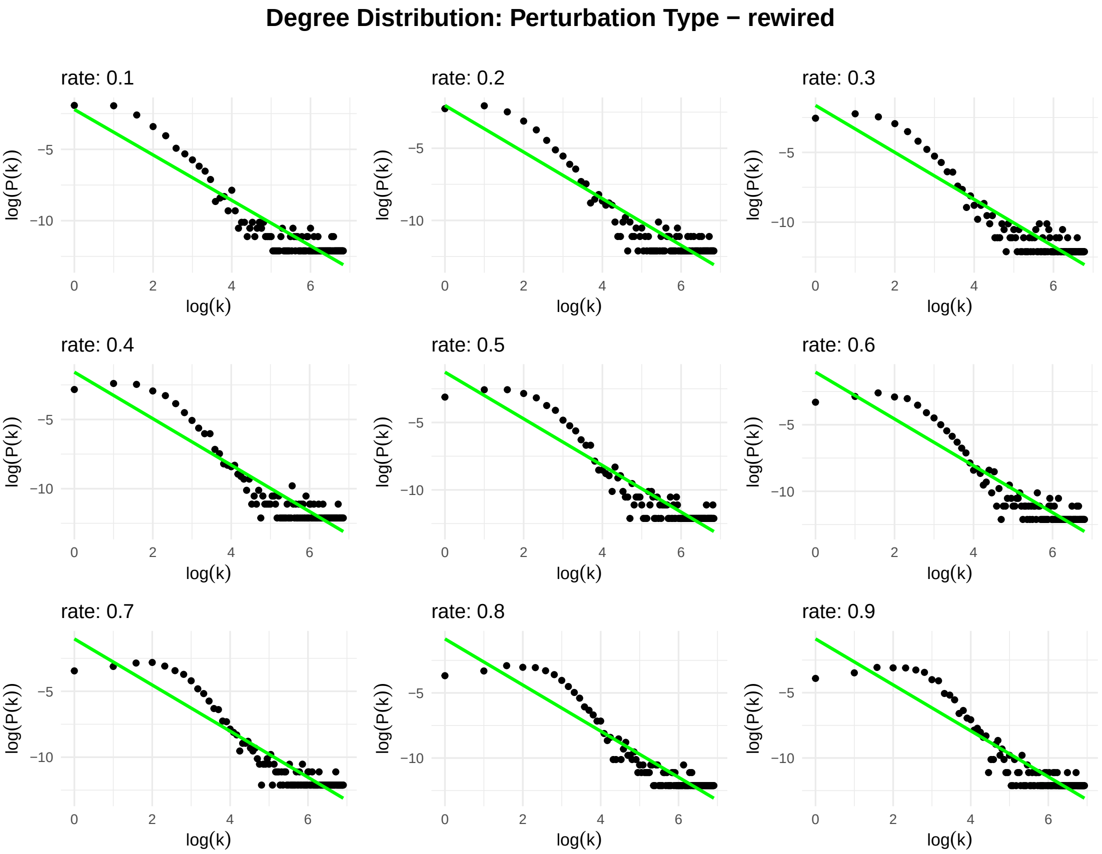

Figure 6: Distribution of degrees in the networks with rewired interactions.

References

[1] Adamic, L. A. and Adar, E. (2003). Friends and neighbors on the web. *Social Networks*, **25**(3), 211–230.

[2] Ashburner, M., Ball, C. A., Blake, J. A., Botstein, D., Butler, H., Cherry, J. M., Davis, A. P., Dolinski, K., Dwight, S. S., Eppig, J. T., *et al.* (2000). Gene ontology: tool for the unification of biology. *Nature Genetics*, **25**(1), 25–29.

[3] Badea, L., Herlea, V., Dima, S. O., Dumitrascu, T., Popescu, I., *et al.* (2008). Combined gene expression analysis of whole-tissue and microdissected pancreatic ductal adenocarcinoma identifies genes specifically overexpressed in tumor epithelia-the authors reported a combined gene expression analysis of whole-tissue and microdissected pancreatic ductal adenocarcinoma identifies genes specifically overexpressed in tumor epithelia. *Hepato-gastroenterology*, **55**(88), 2016.

[4] Brun, C., Chevenet, F., Martin, D., Wojcik, J., Guénoche, A., and Jacq, B. (2003). Functional classification of proteins for the prediction of cellular function from a protein-protein interaction network. *Genome Biology*, **5**, 1–13.

[5] Consortium, G. O. (2019). The gene ontology resource: 20 years and still going strong. *Nucleic Acids Research*, **47**(D1), D330–D338.

[6] Greenfield, A., Madar, A., Ostrer, H., and Bonneau, R. (2010). DREAM4: Combining genetic and dynamic information to identify biological networks and dynamical models. *PloS one*, **5**(10), e13397.

[7] Jaccard, P. (1902). Lois de distribution florale dans la zone alpine. *Bulletin de la Société vaudoise des sciences naturelles*, **38**, 69–130.

[8] Jiang, J. J. and Conrath, D. W. (1997). Semantic similarity based on corpus statistics and lexical taxonomy. In K.-J. Chen, C.-R. Huang, and R. Sproat, editors, *Proceedings of the 10th International Conference on Research in Computational Linguistics*, pages 19–33, Taipei. The Association for Computational Linguistics and Chinese Language Processing.

[9] Lin, D. (1998). An information-theoretic definition of similarity. In J. W. Shavlik, editor, *Proceedings of the Fifteenth International Conference on Machine Learning*, pages 296–304, San Francisco. Morgan Kaufmann Publishers Inc.

[10] Nazor, K. L., Altun, G., Lynch, C., Tran, H., Harness, J. V., Slavin, I., Garitaonandia, I., Müller, F.-J., Wang, Y.-C., Boscolo, F. S., *et al.* (2012). Recurrent variations in dna methylation in human pluripotent stem cells and their differentiated derivatives. *Cell Stem Cell*, **10**(5), 620–634.

[11] Prasad, T. K., Kandasamy, K., and Pandey, A. (2009). Human protein reference database and human proteinpedia as discovery tools for systems biology. *Reverse Chemical Genetics: Methods and Protocols*, pages 67–79.

[12] Resnik, P. (1995). Using information content to evaluate semantic similarity in a taxonomy. In C. S. Mellish, editor, *Proceedings of the 14th International Joint Conference on Artificial Intelligence*, volume 1, page 448–453, San Francisco. Morgan Kaufmann Publishers Inc.

[13] Schaffter, T., Marbach, D., and Floreano, D. (2011). GeneNetWeaver: in silico benchmark generation and performance profiling of network inference methods. *Bioinformatics*, **27**(16), 2263–2270.

[14] Schlicker, A., Domingues, F. S., Rahnenführer, J., and Lengauer, T. (2006). A new measure for functional similarity of gene products based on gene ontology. *BMC Bioinformatics*, **7**(302), 1–16.

[15] Wang, J. Z., Du, Z., Payattakool, R., Yu, P. S., and Chen, C.-F. (2007). A new method to measure the semantic similarity of go terms. *Bioinformatics*, **23**(10), 1274–1281.

[16] Wurmbach, E., Chen, Y.-b., Khitrov, G., Zhang, W., Roayaie, S., Schwartz, M., Fiel, I., Thung, S., Mazzaferro, V., Bruix, J., *et al.* (2007). Genome-wide molecular profiles of hcv-induced dysplasia and hepatocellular carcinoma. *Hepatology*, **45**(4), 938–947.

[17] Yamada, T., Goto, S., and Kanehisa, M. (2004). Extraction of phylogenetic network modules from prokayrote metabolic pathways. *Genome Informatics*, **15**(1), 249–258.
